# Physicochemical Characterization of Polysaccharide–Protein Carriers with Immobilized Yeast Cells Obtained Using the Freeze-Drying Technique

**DOI:** 10.3390/foods13223570

**Published:** 2024-11-08

**Authors:** Nataša Obradović, Bojana Balanč, Ana Salević-Jelić, Mina Volić, Verica Đorđević, Mirjana Pešić, Viktor Nedović

**Affiliations:** 1 Faculty of Technology and Metallurgy, University of Belgrade, Karnegijeva 4, 11120 Belgrade, Serbia; vmanojlovic@tmf.bg.ac.rs; 2 Innovation Center of Faculty of Technology and Metallurgy, University of Belgrade, Karnegijeva 4, 11000 Belgrade, Serbia; bisailovic@tmf.bg.ac.rs (B.B.); mvolic@tmf.bg.ac.rs (M.V.); 3 Faculty of Agriculture, University of Belgrade, Nemanjina 6, 11080 Belgrade, Serbia; ana.salevic@agrif.bg.ac.rs (A.S.-J.); mpesic@agrif.bg.ac.rs (M.P.); vnedovic@agrif.bg.ac.rs (V.N.)

**Keywords:** immobilization, polysaccharide-protein systems, brewery yeast, freeze-drying, food industry, beer production

## Abstract

New techniques for the immobilization of yeast cells have the potential for enhancement of the beer production process. Alongside conventional materials for cell immobilization, there is a rising trend toward polysaccharide–protein systems. This study focused on the immobilization of yeast cells (*Saccharomyces pastorianus*) via a freeze-drying process. The whey protein isolate, sodium alginate, maltodextrin, inulin, and their blends were used for carrier preparation. The effect of a 1.0% inulin solution as a cryoprotectant on the viability of the yeast cells after the freeze-drying process was also analyzed. The powders were assessed for cell viability, moisture content, water activity, solubility, particle size, and surface charge. According to the results, the addition of whey proteins reduced the moisture content, while solubility did not significantly decrease. Samples containing whey protein showed slight diameter variations. The negative surface charge observed in all samples, especially the control, indicates a cell’s tendency to aggregate, demonstrated by optical microscopy. SEM micrographs showed successful cell immobilization in polysaccharide–protein carriers. Furthermore, inulin and whey protein addition enhanced cell protection during the immobilization of cells. The freeze-drying technique demonstrates efficacy in immobilization of yeast cells, indicating its potential for applications in the food and beverage industry.

## 1. Introduction

In recent decades, due to the need for an innovative, continuous, and cost-effective beer production process, the brewing industry has investigated the application of immobilized cell systems. Immobilization of yeast cells has advantages for industrial fermentation processes compared to traditional batch free-cell systems. These advantages include cell protection during fermentation, high cell densities, a lower risk of contamination, the possibility for continuous production, and reusability [1,2,3,4,5,6,7,8].

The extrusion technique is the most frequently employed method for yeast immobilization [9,10,11]. This method is restricted by the challenges of integrating these technologies into industrial practices for large-scale production. The electrostatic extrusion technique can be used to produce carriers with diameters in the range from 0.2 to 2.0 mm. The polymeric carrier matrix’s internal mass transfer limitations are critical factors to consider when selecting an appropriate immobilization technique and carrier material [12,13]. Immobilization of yeast cells can improve the efficiency of the larger production process and enhance the fermentation process. Yeast cells immobilized in Ca–alginate hydrogel carriers, using an electrostatic extrusion technique, notably improved the fermentation process for producing functional craft beer [13]. The immobilization of yeast also has an effect on the production of volatile compounds, including phenols, organic acids, and terpenes, which are responsible for the sensory properties of beer. 

Recent scientific research is focusing on natural and sustainable immobilization techniques alongside traditional methods. An atypical form of biocatalysts for brewing known as yeast “biocapsules” has emerged as a non-expensive, completely natural immobilization technology, where yeast cells adhere to inactive fungal hyphae [14]. The yeast cells remain attached to the hyphae of the fungus, forming spheres with a porous wall that facilitates mass transfer reactions while improving ethanol yields and the aromatic profile of alcoholic beverages [15]. To prevent yeast cell leakage from the “biocapsule” matrix, alginate (0.2% (*w*/*v*)) was used as a coating material for the improvement of fermentation processes for bioethanol production and winemaking [4,16]. The biocapsules could be removed from the fermentation medium as needed, streamlining the process and reducing costs. In winemaking, various cellulosic materials, such as gluten pellets and fruit pieces, have been used for the immobilization of the yeast cells [16]. Natural supports such as apple and quince, which are abundant and low-cost supports with food-grade purity, have been found suitable for continuous processes and contribute to improved sensory qualities in the final products.

Current research is focused on developing new techniques that enable long-term storage and scale-up adaptability in alcoholic beverage production. Spray drying is a technique with broad applicability, but due to the sensitivity of the cells to elevated temperatures, this technique has limitations in yeast cell encapsulation [17]. This technique is cost-effective, and particles can be obtained by spraying the feed solution into a chamber [18]. The freeze-drying process (lyophilization) can be used for thermosensitive bioactive compounds and cells but requires more time in comparison with spray-drying processes [19,20]. Immobilization is an effective technique for the protection of microbial cells from various environmental conditions, but maintaining low manufacturing costs remains a significant challenge in these types of processes. Maltodextrin and alginate are the most used wall materials for cell immobilization via the spray-drying technique [21,22]. Suitable as a carrier in these processes, maltodextrin is a low-cost material with thermoprotective properties, a neutral aroma, and low viscosity at higher concentrations [22,23,24]. Approaches like incorporating sugars, polyols, and proteins into carrier materials have shown potential to improve cell protection during the spray-drying and freeze-drying processes and prolong storage time [25,26]. After the spray-drying procedure, the cell viability and probiotic efficiency were enhanced by the addition of 2.0% sucrose, maltose, and sorbitol to the maltodextrin solution as stress-tolerant components [22,27]. In this context, it was found that oligosaccharides and disaccharides positively impact cell viability during food storage and protect the cellular membrane [25,28]. Many studies have demonstrated that sucrose and trehalose offer protection of microbial cells from temperature, osmotic, and oxidative stress. The inulin addition during the preparation of dough and bread highlighted the significance of this type of prebiotic protector against cell damage during freezing storage [29]. 

However, there is very little information regarding the freeze-drying method for immobilization of yeast cells (*Saccharomyces pastorianus*) in polysaccharide–protein carriers. This study analyzed different materials (sodium alginate, maltodextrin, whey proteins, and inulin) and their blends for cell protection during the immobilization process and storage period. The yeast strain *Saccharomyces pastorianus* W34/70 was immobilized using protein–polysaccharide carriers. The obtained powders were evaluated for cell viability, product yield, moisture content, water activity, solubility, particle size, and ζ potential. The morphological properties of the carriers were evaluated using scanning electron and optical microscopy. The chemical composition of the polysaccharide–protein carriers with immobilized yeast cells was assessed using FT-IR analysis.

## 2. Materials and Methods

### 2.1. Materials

The brewer’s yeast (*Saccharomyces pastorianus W34*/*70*) was cultivated in sterile wort with an 11.0% (*w*/*w*) extract at 25 °C in the shaken flasks. The cells were harvested in the early exponential phase by centrifugation. The yeast strain was purchased from a domestic brewery with headquarters in Belgrade, Serbia. A sodium alginate solution (low viscosity) was prepared by dissolving 1.5 g of sodium alginate powder (Sigma–Aldrich, St. Louis, MO, USA) in 100 mL of distilled water. Whey protein isolate, with an 89.0% protein content based on dry weight and free of artificial colors and flavors, was manufactured by Battery Nutrition, London, UK. Whey protein and maltodextrin 12 (low viscosity, Thermo Scientific Chemicals, Geel, Belgium) powders were dissolved in distilled water to achieve 1.5% (*w*/*v*) of dry matter. An inulin solution was prepared by dissolving 4 g of substance (Sigma–Aldrich, St. Louis, MO, USA) in 100 mL of distilled water.

### 2.2. Preparation of Polysaccharide–Protein/Yeast Suspensions

A protein–polysaccharide/cell suspension was prepared by mixing sodium alginate solution with whey protein isolate solution (ALG-WPI) and maltodextrin with whey protein isolate solution (MAL-WPI) in a ratio of 1:1. Also, sodium alginate–inulin (ALG-INU) and maltodextrin–inulin (MAL-INU) suspensions were evaluated. The final concentration of the inulin in the suspensions was 1.0% (*w*/*v*). The whey protein and maltodextrin solution were pasteurized in a water bath at 60 °C for 60 min. The inoculated medium was incubated at 30 °C for 24 h with continuous shaking. The yeast cell biomass was harvested by centrifugation at 4 °C and 2000× *g* for 20 min. The supernatant was removed, and the biomass was rinsed with saline solution prior to centrifugation. The prepared biomass was weighed and used for immobilization. The yeast cells, at a concentration of 10^9^/mL, were maintained in the sterilized solution until addition to the biopolymer solutions. The yeast concentration in the prepared polysaccharide or polysaccharide–protein suspensions was 8.0% (*w*/*v*). For the control sample, the cell suspension was mixed with a physiological saline solution.

### 2.3. Immobilization of Yeast Cells Using Freeze-Drying Process

The samples (yeast cell suspensions, both with and without carrier materials) were placed in Petri dishes (20 mL) and frozen at −20 °C. After 24 h, the frozen cell dispersions were dried for 24 h at 0.12 mbar using a freeze-dryer (Beta 2–8 LD plus, Christ, Osterode am Harz, Germany). Following the freeze-drying process, the uniform matrices were crushed and homogenized, following the method outlined by [30].

### 2.4. Evaluation of Product Yields

Product yield was calculated as the ratio of the dry weight of the samples obtained after the drying process to the dry weight of the feed suspension, expressed in %.

### 2.5. Cell Viability Before and After the Freeze-Drying Process

One gram of powder with immobilized yeast cells was added to 9 mL of sterile 2.0% trisodium citrate solution. The serial decimal dilutions were prepared in physiological saline. Aliquots of the appropriate dilutions (0.1 mL) were placed on preprepared agar plates with malt extract (Merck KGaA, Darmstadt, Germany). The plates were incubated at 25 °C for 5 days before the yeast colonies were counted. For data analysis, each diluted sample was plated and counted in triplicate. The results are expressed per gram of powder with immobilized cells. The cell viability after the freeze-drying process was calculated from the ratio log ((CFU/g (powder)) and log (CFU/mL (culture in starting suspension)).

### 2.6. Characterization of the Powders with Immobilized Cells

#### 2.6.1. Zeta Potential (ζ) and Carrier Size Distribution

Particle size and zeta potential were monitored using the Malvern Zetasizer Nano ZS (Malvern Instruments, Worcestershire, United Kingdom) with a He–Ne laser at 25 ± 0.1 °C. The powder samples were diluted with distilled water (pH of 6.5) to an approximate concentration of 0.01 g/mL, and for the analyses were taken as fold DTS 1070 cells, followed by sonification. The samples were measured three times, and the results are reported as a mean value with standard deviation.

#### 2.6.2. Water Activity

The as-produced samples filled into plastic disposable sample trays (Novasina AG, Lachen, Switzerland) were placed in the measuring chamber in the device and measured for Aw at ambient temperature until a stable value was reached. The water activity (Aw) values of the samples were measured using a water activity measurement device (LabSwift-aw, Novasina AG, Switzerland).

#### 2.6.3. Moisture Content

The average moisture content in the powder samples with immobilized cells was measured using gravimetrical analysis. Powder samples (0.2 g) were placed in a laboratory oven and dried at 105 ± 2 °C until a constant weight was achieved (approximately 24 h). After this period, the samples were weighed, and their masses were compared to the initial values [31]. The moisture content was calculated from the ratio of the final and initial mass of the samples. The experiments were conducted in triplicate.

#### 2.6.4. Solubility Analysis

The freeze-dried powder samples were reconstituted in distilled water at room temperature and centrifuged at 3000× *g* for 5 min. The supernatant was transferred to pre-weighed Petri dishes and dried in a Memmert oven at 105 °C for 5 h. The solubility (%) was calculated as the ratio of the total dried mass before centrifugation and the mass that remained in the supernatant after centrifugation.

#### 2.6.5. Fourier Transform Infrared Spectroscopy

Fourier transform infrared (FT-IR) spectroscopy was applied to evaluate the structural properties of the obtained powders and the chemical interactions between their constituents. The prepared samples were subjected to the analysis in attenuated total reflection mode (ATR) in the wavenumber range of 4000–600 cm^−^^1^ with a resolution of 4 cm^−^^1^ and 100 accumulations per scan using an IRAffinitty-1S (Shimadzu, Kyoto, Japan).

#### 2.6.6. Optical Microscopy

The microscopic analyses were conducted to verify the particle size, shape, and potential cell aggregations. The samples were examined using a digital light microscope Motic BA210 Series (Hicksville, New York, NY, USA) equipped with an image analyzer (Motic Images Plus 2.0 ML, Hicksville, New York, NY, USA) at 40× magnification.

#### 2.6.7. Analysis of the Carrier’s Surface and Microstructure Morphology Using Scanning Electron Microscopy

Scanning electron microscopy was used to evaluate the surface morphology of the powder samples. The freeze-dried samples were coated with gold/platinum alloy in a vacuum employing a fine ion sputter coating process. The powder samples were examined using a TESCAN MIRA 3 XMU (Cranberry Township, PA, USA) field-emission scanning electron microscope at an accelerating voltage of 10 kV.

### 2.7. Storage Stability

The powder samples with the best cell viability and production yield after the freeze-drying process were stored under refrigerated conditions at 4 °C, and after 28 days, cell viability in the samples was assessed.

### 2.8. Statistical Analysis

The results were analyzed using one-way ANOVA, with significant differences between the samples determined by the Tukey test (*p* < 0.05). The analyses were performed using Microsoft Excel (Microsoft Office 365 Edition, Redmond, WA, USA) and Origin Pro 8.5 (Origin Lab Corporation, Northampton, MA, USA). All measurements were performed in triplicate.

## 3. Results and Discussion

### 3.1. Impact of the Carrier Composition on the Cell Viability, Product Yield, Water Activity, and Moisture Content Following the Freeze-Drying Process

Lyophilization, or freeze-drying, is an important procedure for the preservation of yeast cells. The survival rates and cell viability after freeze-drying are influenced by factors such as cell size, the capacity to tolerate osmotic stress, yeast strain and the utilization of cryoprotectants. After the drying process, desiccated cells that retain their viability result in the ability to rehydrate with minimal damage. Among the most critical factors for the survival rate and fermentation efficacy of freeze-dried yeast starters are protective agents [32,33]. Currently, polysaccharide–protein carriers have not been utilized for the immobilization of *Saccharomyces pastorianus*, nor has the combination of this technique with inulin as a cryoprotectant been explored in the literature. Table 1 presents the immobilization techniques and carriers currently used for this type of yeast strain. The most common techniques used for this type of yeast strain were extrusion, immobilization using filamentous fungus cells and immobilization on the support surface.

According to the results, after the freeze-drying process, the carriers showed a satisfactory product yield. A significantly higher yield (*p* < 0.05) was observed for the powders containing whey protein isolate, particularly in the alginate–whey protein isolate and maltodextrin–whey protein isolate systems, compared to the alginate carriers. For all types of powders, the product yield was above 90% (Table 2). The addition of whey protein isolate and inulin significantly improved cell viability and survival during lyophilization. Maltodextrin demonstrated better protective properties compared to alginate-based powders. Comparable results for the yields were also obtained for the carriers prepared with maltodextrin, sodium caseinate, and corn starch used for the encapsulation of *Saccharomyces cerevisiae* [24]. The protective effects of whey proteins, skimmed milk, and maltodextrin on the probiotic cells (*Lactobacillus rhamnosus* and *Lactobacillus plantarum*) during the spray-dying and freeze-drying processes have been investigated and confirmed by other authors in the literature [17,20,24,25,26]. Additionally, the protective properties of inulin–maltodextrin carriers were evaluated for microencapsulation of bioactive compounds using the spray-drying method [41]. The results indicate that the carrier materials significantly improved cell viability compared to free cells (control samples) during the immobilization process, particularly ALG-INU and MAL-WPI (Table 2).

Water activity is an important parameter to define the microbial stability of the samples by showing the amount of free water available. For instance, the growth of pathogenic bacteria is not possible when Aw values are under 0.85–0.86. Conversely, yeasts and molds are usually absent when Aw values are below 0.62 [42]. The Aw values for the powder samples ranged from 0.19 to 0.27 and are shown in Table 2. According to the results, there were no significant differences among the samples, suggesting that the addition of protein or inulin did not affect the Aw values of the powders. The low Aw values indicate good storage and physical stability of the tested samples. After the solubility analysis, the polysaccharide carriers showed slightly better solubility compared to those containing whey proteins. The solubility of the polysaccharide–protein carriers in distilled water was ~93%. There was no statistically significant difference (*p* > 0.05) in solubility with the addition of protein or inulin.

After 28 days of storage, cell survival in ALG-based powders was 58.02 ± 1.84%, while in the powder ALG-WPI, it was not significantly higher at 62.48 ± 2.01. In the MAL-based powders, cell survival was significantly higher, at 80.12 + 2.86 and 83.69 ± 1.18 for the MAL and MAL-WPI samples, respectively. The addition of inulin did not have a significant influence on the survival of the cells after 28 days. Free cell survival after this period was 56.13 ± 3.6%, indicating that maltodextrin-based powders provided better protection compared to alginate-based during storage. The satisfactory cell viability after the storage period can be attributed to favorable water activity parameters and the moisture content of the powders [20].

### 3.2. Characterization of the Powders with Immobilized Cells: Size, Morphological, and Surface Properties

**Figure 1:**
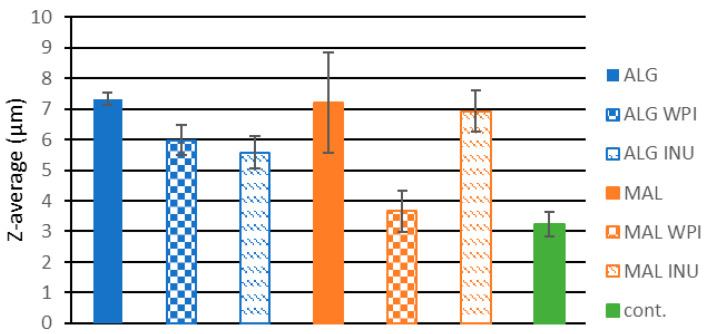
Z-average size of the carriers with immobilized cells and a control sample with free (non-immobilized cells).

The size of the biocatalysts increased with the addition of carrier materials, compared to free cells. The results are displayed in Figure 1. The distribution is broad for all samples and the average values are between 3.5 and 7.4 µm for the powders with immobilized cells. The dried free cells had the smallest particle size, measuring approximately 3.2 µm. The addition of whey protein to polymers slightly reduced the average size of the powders. It is known that the WPI has emulsifying properties and reduces surface tension, causing the creation of smaller particles [43]. The addition of inulin did not cause significant changes in the average size of the particles and similar findings were reported by [44], who investigated the effect of chitosan–alginate encapsulation with inulin on the survival of *Lactobacillus rhamnoses.*

**Figure 2:**
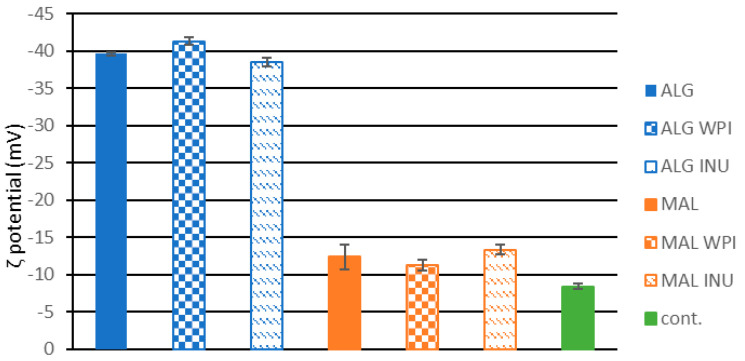
Zeta-potential of the carriers with immobilized cells and a control sample with free (non-immobilized cells).

Zeta (ζ) potential represents the surface charge of the colloidal particles, indicating the polymer interactions in the particles and their tendency toward an agglomeration. If the zeta-potential values are equal to or greater than 30 mV (in absolute value), they are considered stable. In this study, zeta potential was determined for both free cells and cells coated with polymers/proteins dispersed in distilled water. All samples exhibited negative surface charge values. Samples prepared with alginate had a zeta potential between −38.5 and −41.3 mV, implying a great repulsive force between the colloidal particles and their good stability (Figure 2).

**Figure 3:**
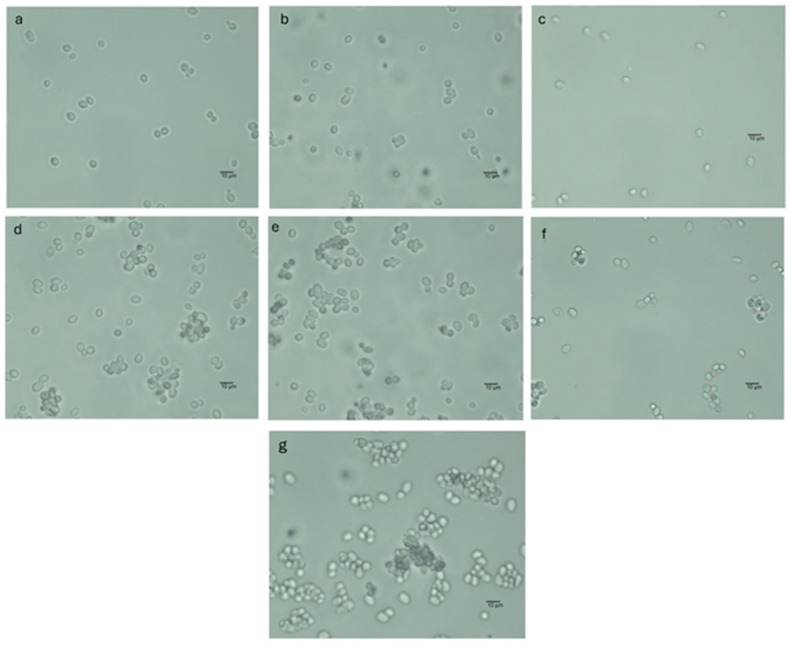
Carriers with immobilized yeast cells (**a**) ALG-yeast cells, (**b**) ALG-WPI-yeast cells, (**c**) ALG-INU-yeast cells, (**d**) MAL-yeast cells, (**e**) MAL-WPI yeast cells, (**f**) MAL-INU-yeast cells, and (**g**) control samples with free cells assessed by optical microscopy (magnification 40×), scale bar is 10 µm.

The addition of inulin or WPI did not influence this parameter. Samples where maltodextrin was used also had a negative charge with slightly lower values (between −11.3 and −13.3 mV), which is in accordance with literature data for maltodextrin-dried particles [45,46,47]. The addition of inulin did not influence the ζ potential of the carriers. The reduction in zeta potential in systems like MAL-WPI (maltodextrin–whey protein isolate), MAL (maltodextrin), and MAL-INU (maltodextrin–inulin) may be ascribed to the interactions between the carrier components in a manner that neutralizes these surface charges. The interaction between WPI or inulin, which have pH-dependent charges, and maltodextrin may result in a diminished overall surface charge. A decrease in zeta potential may indicate that the polysaccharides or proteins are well adsorbed onto the particle surfaces as an efficient coating rather than ineffective biopolymer interactions [45,46,47]. The obtained values for maltodextrin surface charge align with the images from the optical microscope and the tendency of cells to agglomerate in comparison with ALG-based systems (Figure 3).

Carriers with immobilized cells were also analyzed under an optical microscope, and the control samples showed a tendency to agglomerate. This was further confirmed by the surface charge results of the free cells. The zeta potential of the free cells was the lowest among all analyzed samples, −8.44 ± 0.21 mV (Figure 2). The optical microscope image highlights the ability of alginate to reduce cell agglomeration (Figure 3). The affinity of the yeast cells (*S. pastorianus*) for the agglomeration was also observed in the systems containing nanoparticles [48]. The yeast cell wall is primarily composed of fibrous β-1,3 glucan and mannoproteins, which contribute to the anionic surface charge of yeast cells. Consequently, electrostatic interactions may also influence cell-to-cell binding forces and the agglomeration of the cells [49].

**Figure 4:**
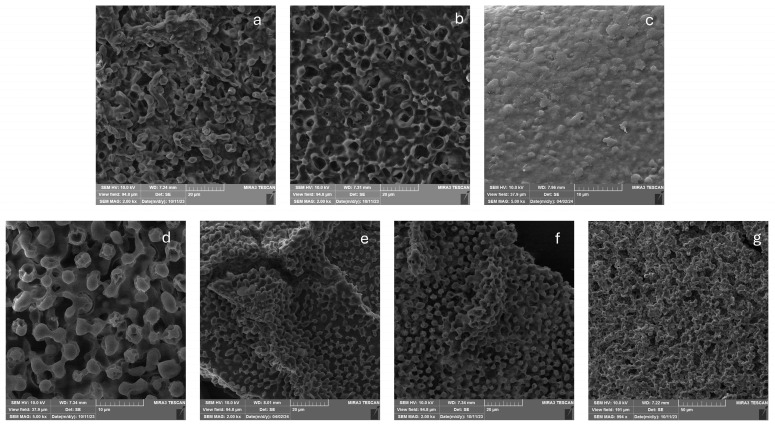
SEM micrographs of (**a**) ALG-yeast, (**b**) ALG-WPI-yeast, (**c**) ALG-INU-yeast, (**d**) MAL-yeast, (**e**) MAL-INU-yeast, (**f**) MAL-WPI-yeast powders and (**g**) free yeast cells.

SEM micrographs were used to analyze the surface morphology of the polysaccharide–protein carriers with magnifications of 2000× and 5000×. Figure 4 shows the successful immobilization of yeast cells, which are randomly distributed throughout the carrier. The immobilized yeast cells in the alginate carriers were also analyzed using SEM micrography in the literature [13]. In Figure 4a,b, the systems with added whey proteins show the greater cell cover by the carrier material, which could be explained by the cells’ affinity for nutrient-enriched materials. In Figure 4, cells are located on the surface and within the cavities of the carriers. All types of carriers exhibit an irregular shape and a dense microstructure with randomly distributed immobilized cells. The carriers with WPI and inulin have less visible cracks on the surface. The existence of fractures can be associated with the rate of water evaporation throughout the process [50]. The microstructure of the complex coacervate indicates that whey protein and inulin diminished the imperfections, leading to a uniform material for yeast protection.

The cell viability after the freeze-drying process aligned with the cell affinity for the carriers demonstrated in Figure 4c,e,f, particularly for ALG-INU, MAL-WPI, and MAL-INU. The addition of WPI and inulin resulted in a smoother carrier surface. A combination of proteins and polysaccharides raises the glass transition temperature compared to carbohydrates alone, thereby stabilizing the carrier and enhancing cell viability [51]. The impact of whey protein addition on the surface of biopolymer carriers was also noted in the systems with encapsulated starter culture [20]. Free cells in the control sample are shown in Figure 4g.

### 3.3. Spectroscopic Analysis of the Powders with Immobilized Cells

**Figure 5:**
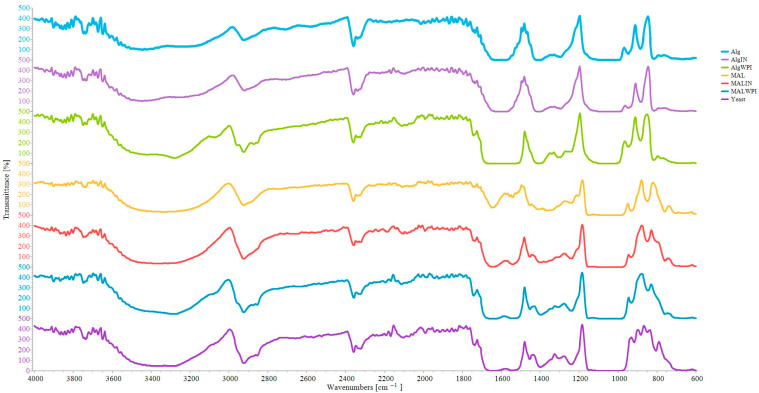
FT-IR spectra of the yeast cells and powders with immobilized cells.

The characteristic functional groups, interactions between the carrier components, and immobilized yeast cell identification were analyzed using FT-IR spectroscopy. Figure 5 shows the spectra of freeze-dried yeast cells and the corresponding immobilized cells. As one can observe, the bands of the yeast and carriers mainly overlapped. All spectra presented a broad band in the range ~3600–3000 cm^−^^1^, corresponding to stretching vibrations of free, inter, and intramolecular -OH groups. Within this range, a shoulder at ~3070 cm^−^^1^ was observed at the spectra of ALG-WPI and MAL-WPI, most probably originating from the amide bands of WPI. The bend at the spectrum of the yeast at ~2920 cm^−^^1^ can be assigned to lipids of the plasmatic membrane. This bend was present in the spectra of the carriers as well and can also be attributed to C-H stretching vibrations of aliphatic groups of alginate and maltodextrin, being contributed to by the vibrations of WPI [52,53]. The peaks around 2930 cm^−1^ for alginate/whey/probiotic culture carriers were also used to indicate the presence of encapsulated cells in the hydrogel carriers [54].

The characteristic bands in the yeast spectrum originating from proteins were found at 1645 (Amide I, C=O stretching vibrations), 1541 (Amide II, N-H and C-N stretching vibrations), and in the range ~1400–1240 cm^−^^1^ (Amide III, N-H bending and C-N stretching vibrations of amide bonds and CH_2_ groups wagging vibrations) [51,54]. Interesting findings within this region were found in the spectra of the immobilized yeast cells. The spectra of ALG and ALG-INU systems showed a band at ~1600 cm^−^^1^, which was present at ~1650 cm^−^^1^ in the spectra of MAL and MAL-INU, being assigned to asymmetric COO- group vibrations [53].

A shift of this bend to ~1620 and 1640 cm^−^^1^ was observed at ALG-WPI and MAL-WPI spectra, respectively, which may be ascribed to water residue absorption [55], Amide I of WPI, or potential interactions between polysaccharides and WPI. The band originating from Amide II of the yeast cells was present only in the spectra of MAL-based carriers. The carriers showed a band at ~1410–1400 cm^−^^1^ due to symmetric COO-group vibrations [53]. The bands representing immobilized yeast cells, including amide III of proteins, phosphorylated proteins, and phospholipids, were observed in region ~1360–1240 cm^−^^1^ in the spectra of the immobilized systems [40]. These types of bands confirm the efficient immobilization of yeast cells.

Another region present in the spectrum of yeast was in the range of ~1200–800 cm^−^^1^, originating from carbohydrates, such as β-glucans, glycogen, and mannans [52]. In this region, the employed carbohydrate matrices showed C-O-C stretching vibration bands [53]. The band in the yeast spectrum at 1040 cm^−^^1^ ascribed to glycogen was found at approximately 1030 and 1020 cm^−^^1^ in the spectra of ALG-based and MAL-based systems, respectively [56]. The carriers with immobilized cells also showed bands at ~800–750 cm^−^^1^ (C-H in-plane bending) from the polysaccharides [53]. Altogether, the FT-IR spectroscopy analysis indicated that the yeast-originating bands were mainly overlapped by those of the matrices, which can be beneficial for protecting the immobilized cells. Also, the discussed spectra features indicated successful immobilization of the yeast cells within the ALG-based and MAL-based carriers.

## 4. Conclusions

Yeast cells (*Saccharomyces pastorianus*) were immobilized in a polysaccharide–protein carrier using a freeze-drying technique. The systems based on maltodextrin exhibited a high cell survival rate (>83.3%) and excellent product yield (>95%). A cell agglomeration tendency was demonstrated using optical microscopy and highlighted the significance of the carriers for system stability. Spectroscopy analyses confirmed chemical interactions between the polysaccharides and proteins within the carrier structure. SEM micrographs showed successful cell immobilization and smoother carrier surfaces in samples prepared with WPI and inulin. The obtained powders were microbiologically stable based on water activity and moisture content, especially polysaccharide–protein based carriers. Maltodextrin in combination with whey protein isolate and inulin demonstrated the most promising features as a carrier material during the freeze-drying process and storage time, indicating its potential for application in the food and beverage industry.

## Figures and Tables

**Table 1:** Type of the carrier material, technique and physiochemical parameters of the systems with immobilized yeast strain *Saccharomyces pastorianus*.

Type of the Carrier	Immobilization Technique	Physiochemical Parameters	Storage Properties	References
Low-viscosity-grade sodium alginate	Extrusion	Spherical shape with dimensions of 1.50 mm ± 0.13 mm.	-	[34]
Filamentous fungus cells	Immobilization using filamentous fungus cells	Biocapsules size may vary from few millimeters of diameter to several centimeters	-	[35]
Ceramic chamotte tablet	Immobilization on the support surface	The height and diameter of the chamotte tablets were 5 and 15 mm	Systems remained stable after storage for 1 week in the medium for immobilization	[36]
Spent grains and corncobs	Immobilization on the support surface	Particles with a maximum diameter of 0.1 cm	-	[37]
Sodium alginate	Extrusion	Beads with 1.40 mm diameter, mesoporous structure (SEM)	-	[38]
Alginate/chitosan matrix	Extrusion	Microcapsule	-	[39]
Sodium alginate	Extrusion	Bead dimensions were between 1.89 mm ± 0.13 mm, homogeneous structure	-	[40]

**Table 2:** Product yield, moisture content (%), water activity (Aw), and cell viability after the freeze-drying process.

Sample	Water Activity	Cell Viability After the Freeze-Drying Process, %	Product Yield, %	Moisture Content, %
ALG	0.188 ± 0.003 ^a^	78.400 ± 4.410 ^b,c^	90.401 ± 2.101 ^a^	7.50 ± 0.44 ^c^
ALG-WPI	0.220 ± 0.003 ^b^	74.051 ± 3.090 ^b^	96.311 ± 1.361 ^b,c^	6.50 ± 0.31 ^b^
ALG-INU	0.222 ± 0.004 ^b^	84.080 ± 3.360 ^c^	92.202 ± 0.890 ^a^	12.50 ± 0.67 ^d^
MAL	0.253 ± 0.003 ^d^	74.301 ± 2.901 ^b^	95.602 ± 1.100 ^b,c^	7.43 ± 0.34 ^c^
MAL-WPI	0.224 ± 0.001 ^b^	84.477 ± 4.100 ^c^	97.730 ± 0.970 ^c^	4.00 ± 0.12 ^a^
MAL-INU	0.240 ± 0.006 ^c^	83.328 ± 3.010 ^c^	95.901 ± 1.229 ^b,c^	7.00 ± 0.27 ^c^
CONTROL	0.271 ± 0.008 ^e^	66.645 ± 2.683 ^a^	94.793 ± 1.661 ^a,b^	6.34 ± 0.23 ^b^

Different superscripts within the same column indicate a significant difference of means according to Tukey’s honest significant difference (HSD) test (*p* < 0.05).

## Data Availability

The original contributions presented in the study are included in the article, further inquiries can be directed to the corresponding author.
